# Occupational exposure to formaldehyde and risk of non hodgkin lymphoma: a meta-analysis

**DOI:** 10.1186/s12885-019-6445-z

**Published:** 2019-12-23

**Authors:** Simona Catalani, Francesca Donato, Egidio Madeo, Pietro Apostoli, Giuseppe De Palma, Enrico Pira, Kenneth A. Mundt, Paolo Boffetta

**Affiliations:** 1 Department of Medicine and Surgery Specialties, Radiological Sciences and Public Health University of Brescia, 25133 Brescia, Italy; 2 Department of Public Health and Pediatric Sciences, University of Turin, Turin, Italy; 3 Cardno ChemRisk, Boston, MA USA; 4 Tisch Cancer Institute, Icahn School of Medicine at Mount Sinai, New York, NY USA; 5 Department of Medical and Surgical Sciences, University of Bologna, Bologna, Italy

**Keywords:** Non-Hodgkin lymphoma, Formaldehyde, Cancer, Meta-analysis

## Abstract

**Background:**

Formaldehyde, a widely used chemical, is considered a human carcinogen. We report the results of a meta-analyses of studies on the relationship between occupational exposure to formaldehyde and risk of non-Hodgkin lymphoma (NHL).

**Methods:**

We performed a systematic review and meta-analysis according to international guidelines and we identified 12 reports of occupational populations exposed to formaldehyde. We evaluated inter-study heterogeneity and we applied a random effects model. We conducted a cumulative meta-analysis and a meta-analysis according to estimated average exposure of each study population.

**Results:**

The meta-analysis resulted in a summary relative risk (RR) for NHL of 0.93 (95% confidence interval 0.83–1.04). The cumulative meta-analysis suggests that higher RRs were detected in studies published before 1986, while studies available after 1986 did not show an association. No differences were found between different levels of occupational exposure.

Conclusions Notwithstanding some limitations, the results of this meta-analysis do not support the hypothesis of an association between occupational exposure to formaldehyde and risk of NHL.

## Background

Formaldehyde is a high-volume industrial chemical with 5 million tons produced annually in the United States (US). Major occupational sources of exposure include manufacturing of resins for wood products, furniture and fixtures, wearing apparel and textiles, chemicals and plastic products;

occupational exposure to formalin, a solution of formaldehyde in water, occurs in several service industries, including medical, dental and veterinary [1–3].

Exogenous formaldehyde is rapidly metabolized at the site of entry (typically the upper respiratory tract). Formaldehyde is also produced endogenously and is an essential intermediate in the biosynthesis of purines, thymidine, and various amino acids [4].

Exposure to relatively high concentrations (i.e., long-term exposure to concentrations greater than about 4 ppm) formaldehyde has been shown to cause nasal cancer in animal experiments [5, 6], and associations have been reported between occupational exposure to formaldehyde and risk of several types of cancer [7–9].

Human cancer risks associated with formaldehyde exposure have been investigated in occupational cohorts and community-based case–control studies. Occupational cohort studies generally provide higher-quality evidence than population-based case–control studies, primarily due to better exposure data and a greater potential for higher and more sustained levels of exposure [10].

In 2006 the International Agency for Research on Cancer (IARC) concluded that there was sufficient evidence in humans for the carcinogenicity of formaldehyde, based on results of epidemiological studies reporting an association with nasopharyngeal cancer; at that time, IARC also concluded that there was “strong but not sufficient evidence for a causal association between leukaemia and occupational exposure to formaldehyde” [2]. In 2009, IARC added leukaemia to the list of neoplasms caused by formaldehyde, although this was determined by a small majority of the Working Group [11]. Subsequently, the National Toxicology Program of the National Institute of Environmental Health Sciences changed the classification of formaldehyde from “anticipated to be carcinogenic in humans” to “known to be a human carcinogen” [3].

Numerous reviews and meta-analyses have focused on associations between formaldehyde and lymphohematopoietic cancer in general or leukemia [10, 12, 13], but no similar review and meta-analysis has been published on epidemiological studies evaluating exposure to formaldehyde and risk of non-Hodgkin lymphoma (NHL).

NHL is a histologically and genetically heterogeneous group of malignancies originating from B- and T-cell lineages that account for 2.7% of the global cancer burden, with important variations in geographic and temporal patterns of incidence [14, 15]. Known causes of NHL explain only a small proportion of cases which occur globally; these include chemotherapy treatment or severe immune system dysregulation, infection with Epstein–Barr virus, hepatitis C virus, and Helicobacter pylori, as well as autoimmunity and atopic conditions, high BMI, and tobacco smoking in addition to family history of lymphatic neoplasms [16].

We therefore conducted a systematic review and meta-analysis of epidemiological studies published investigating the association between occupational exposure to formaldehyde and risk of NHL. We excluded studies based on either environmental or dietary exposure to formaldehyde because of lower and less precisely defined exposure levels compared to workplace exposure.

## Methods

The systematic review and meta-analysis were performed according to the guidelines specified in the PRISMA-statement [17]. The methods were documented in a protocol (available upon request); the PRISMA checklist is included in Additional file 1: Table S1.

### Literature search and study selection

We conducted comprehensive literature searches of Scopus and PubMed, up to 12 July 2018; PubMed “related article” links and reference lists of key studies and reviews were used to complement the primary searches. The searches included the keywords (“formaldehyde”) AND (“cancer” OR “neoplasm” OR “lymphoma” OR “non-Hodgkin lymphoma”).

To be included in the meta-analysis, studies had to fulfill the following criteria: (i) original reports based on workers exposed to formaldehyde; (ii) studies in which the results were reported for NHL ([ICD-9 codes 200, 202]: lymphosarcoma and reticulosarcoma and other specified malignant tumors of lymphatic tissue; other malignant neoplasms of lymphoid and histiocytic tissue and [ICD-10 codes C82, C85]: follicular lymphoma; other specified and unspecified types of non-Hodgkin lymphoma), alone or with other categories of lymphohematopoietic neoplasms (e.g., Hodgkin lymphoma [ICD-9 code 201] or multiple myeloma [ICD-9 code 203]), excluding leukemia; (iii) studies in which a measure of association between formaldehyde exposure and risk of NHL, expressed either as standardized mortality ratios (SMR), standardized incidence ratios (SIR), proportionate mortality ratio (PMR), relative risk (RR) or odds ratio (OR) either was reported or could be derived from the publication.

**Fig. 1:**
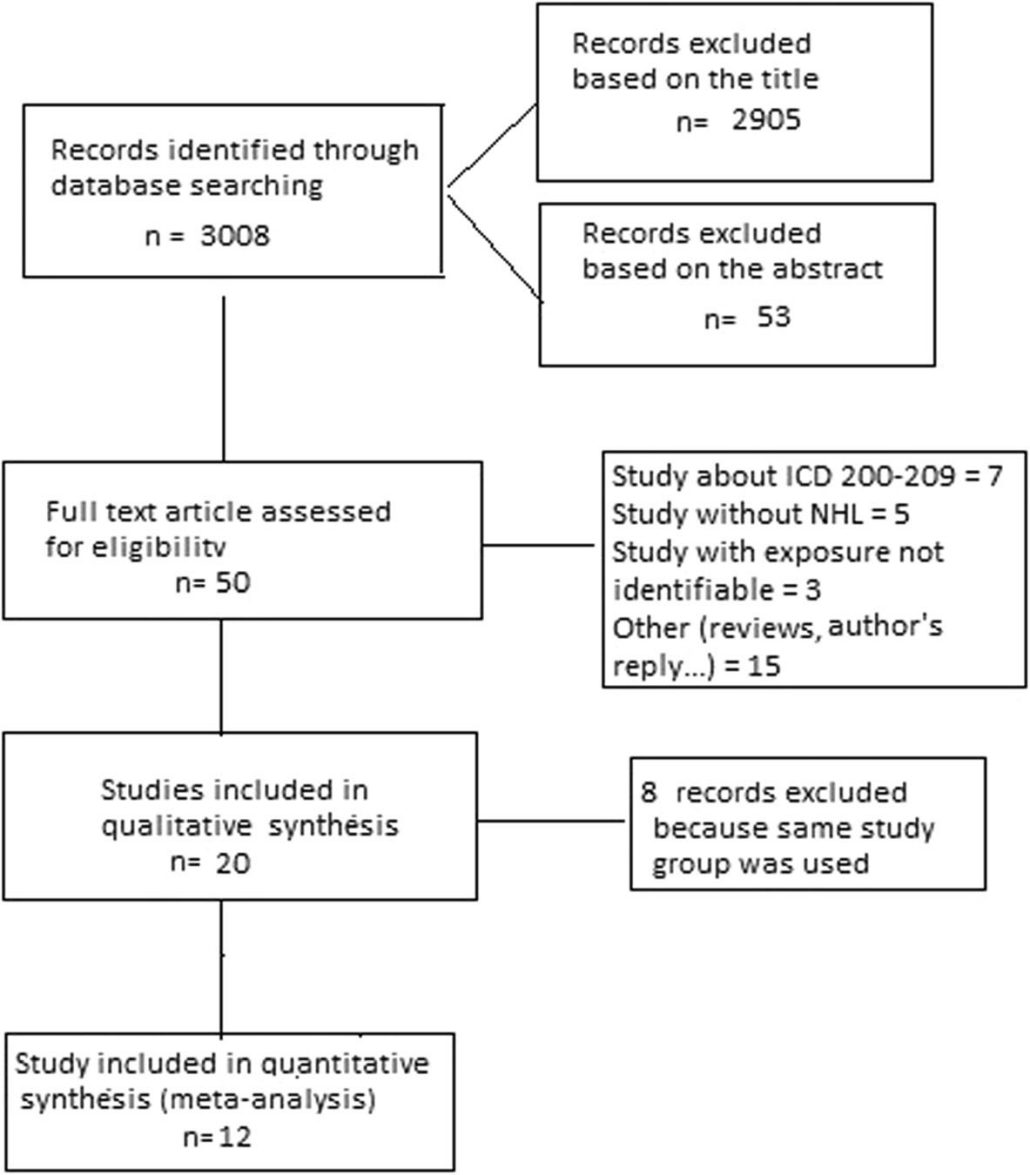
Flow chart of search and selection of studies included in the review and meta-analysis

Two authors (SC, FD) independently reviewed the list of titles and abstracts, to determine which studies potentially met the inclusion criteria. Duplicates and irrelevant references were eliminated. The final selection was based on the examination of the full text of potentially relevant articles. Cases of disagreement or doubt were resolved with the inclusion of a third author (PB). The search and selection process are shown in Fig. 1.

After reviewing the titles of 3008 articles, we eliminated 2905 that did not appear to be relevant, and reviewed the abstracts of the remaining 103 articles. Review of these led to the elimination of 53 articles that did not meet the inclusion criteria, leaving 50 articles for detailed review. Thirty of the 50 articles subsequently were eliminated because exposure was not clearly defined, or results were reported for all lymphohematopoietic neoplasms combined ([ICD9-codes 200–209] malignant neoplasm of lymphatic and hematopoietic tissue). Among the remaining 20 articles, some referred to the same study population; in such cases, we selected the report with the most complete information (i.e., longest follow-up), leaving for the meta-analysis 12 reports of studies of non-overlapping populations (Table 1) [18–29].

**Table 1:** Selected characteristics of studies included in the meta-analysis

Reference	Country	Study design	Study period*	Population	Levels of Exposure	Outcome	I/M	Obs/Exp	N**	% men
Walrath et al., 1983 [18]	USA, New York	Co	1902–1980 /1925–1980 (death)	Embalmers	0.20 to 0.91 ppm	ICD-8200; 202,203	M	11/9.6	n.a.	100
Walrath et al., 1984 [19]	USA, California	Co	1916–1978/1925–80 (death)	Embalmers	0.2 and 0.9 ppm	ICD-8200; 202,203 208 209	M	7/6.1	n.a.	100
Stroup et al., 1986 [20]	USA	Co	1888–1969	Anatomist	cumulative exposure	ICD-8200; 202, 203, 208–209	M	8/5.9	2% + 3% migrated	100
Partanen et al., 1993 [21]	Finland	case-referent nested	1957–1982	Wood Industry	reconstructed using a plant/period-specific job exposure	ICD-7200, 202	M	4/n.a.	n.a.	
Hansen et al., 1995 [22]	Denmark	record linkage	1970–1984	Facilities that used or manufactured > 1 kg per year of formaldehyde	Job title in formaldeide associated facilities	ICD-7200,202	I	32/37.5		
Stellman et al., 1998 [23]	USA	Co	1982--f.up 1988	Wood Dust Exposure	check.off list	ICD-9200,202	M	11/n.a.	2%	100
Band et al., 1997 [24]	Canada	Co	1950–1992	pulp and paper mill	–	ICD-9200, 202	M	35/n.a.	10%	100
Pinkerton et al., 2004 [25]	USA	Co	1955-f.up 1998	3 garment industries	personnel records of exposure	ICD-9200; 202,203	M	33/n.a.	n.a	18.3
Meyers et al., 2013 [26]	USA	Co	1955/59–2008	formaldehyde resins	campaign of personal sampling	ICD-10 (C46.3,C82-C85,C88.0,C88.3,C91.4,C96)	M	66/n.a	1.1	18
Pira et al., 2014 [27]	Italy	Co	1930–1966 or 1934–58 until 2004	laminated plastic workers	time of employment	ICD-9200–202	M	4/5.4	3.1	81
Coggon et al., 2014 [28]	UK	Co	1941–2012	chemical workers	recorded titles of jobs	ICD-9200,202	M	13/14.4		100
Checkoway et al., 2015 [29]	USA	Co	1930–1966 or 1934–58 until 2004	manufacturersor users of formaldehyde,	estimated for each job from individual work histories	ICD-8200,202	M	94/n.a.		81.8

*NR* not relevant, *NA* not available, *Co* cohort study, *CC* case-control study, *NCC* case-control study nested in a cohort, *I* incidence, *M* mortality, *N* number of cohort members (cohort studies) or number of cases (case-control studies), *NHL* non-Hodgkin lymphoma, *ICD* International Classification of Diseases

Five of these 12 reports [29–33] were based on a single large cohort study of workers from 10 US plants producing or using formaldehyde. This study was initiated by the US National Cancer Institute (NCI) in the early 1980s in collaboration with the Formaldehyde Institute, and the first results were published in 1986 [30]. We included in the meta-analysis the results of the most recent analysis of this cohort [29]. Similarly, we selected the article by Pinkerton and coworkers (2004) [25], the most recent of three analyses of a cohort of workers from three garment manufacturing facilities, located in Georgia and Pennsylvania, USA [25, 34, 35], and the article by Coggon and colleagues (2014) [28], the most recent of three articles based on a cohort of workers from six chemical factories in England and Wales [28, 36, 37].

### Data extraction

We extracted key characteristics of each of the studies retained for the meta-analysis (Table 1). We aimed at investigating NHL (i.e., International Classification of Diseases, version 9 (ICD-9) codes 200, 202 and ICD-10 codes C82, C85); however, results from some studies were available only for different disease categories (see Table 1 for details). When results were reported based on different strategies for adjustment for potential confounders, we included the fully adjusted risk estimates. When the data were reported for different levels of exposure, we chose the category at highest exposure.

### Data analysis

We evaluated inter-study heterogeneity using the general variance-based method, and applied a random effects model which incorporates between-study variation into the summary variance estimate to estimate summary RR and 95% confidence interval (CI) estimates [38].

We conducted sensitivity analyses excluding one study at a time from the meta-analysis, and a cumulative meta-analysis according to the year of publication of the individual studies. Finally, we conducted a meta-analysis according to the type of average exposure level of the workers included in each study, categorized as very low, low and medium-high exposure.

The exposure in each study was classified as very low when the levels of formaldehyde was estimated lower than 10 μg/m^3^, low when it was between 10 and 200 μg/m^3^ and medium-high when it was higher than 100 μg/m^3^.

The classification was made on the basis of the data on exposure levels available from the studies included in the meta-analysis and on the type of industry; it does not aim to be comprehensive, such as the one proposed by IARC [2].

We generated funnel plots of the results included in the meta-analysis and used the test proposed by Egger et al. [39] to assess possible publication bias.

## Results

The meta-analysis comprised results from 12 independent studies [18–29], only the study by Hansen et al. [22] analyzed the incidence of NHL, while the remaining studies were based on mortality data. Overall, these studies included a total of 318 NHL cases or deaths.

Risk estimates for NHL (ICD-9200,202) were reported in 7 studies [21–24, 26, 28, 29]; in 5 additional studies [18–20, 25, 27] we combined, using a fixed-effect model, the results calculated for different ICD-9 categories, as shown in Table 1.

**Fig. 2:**
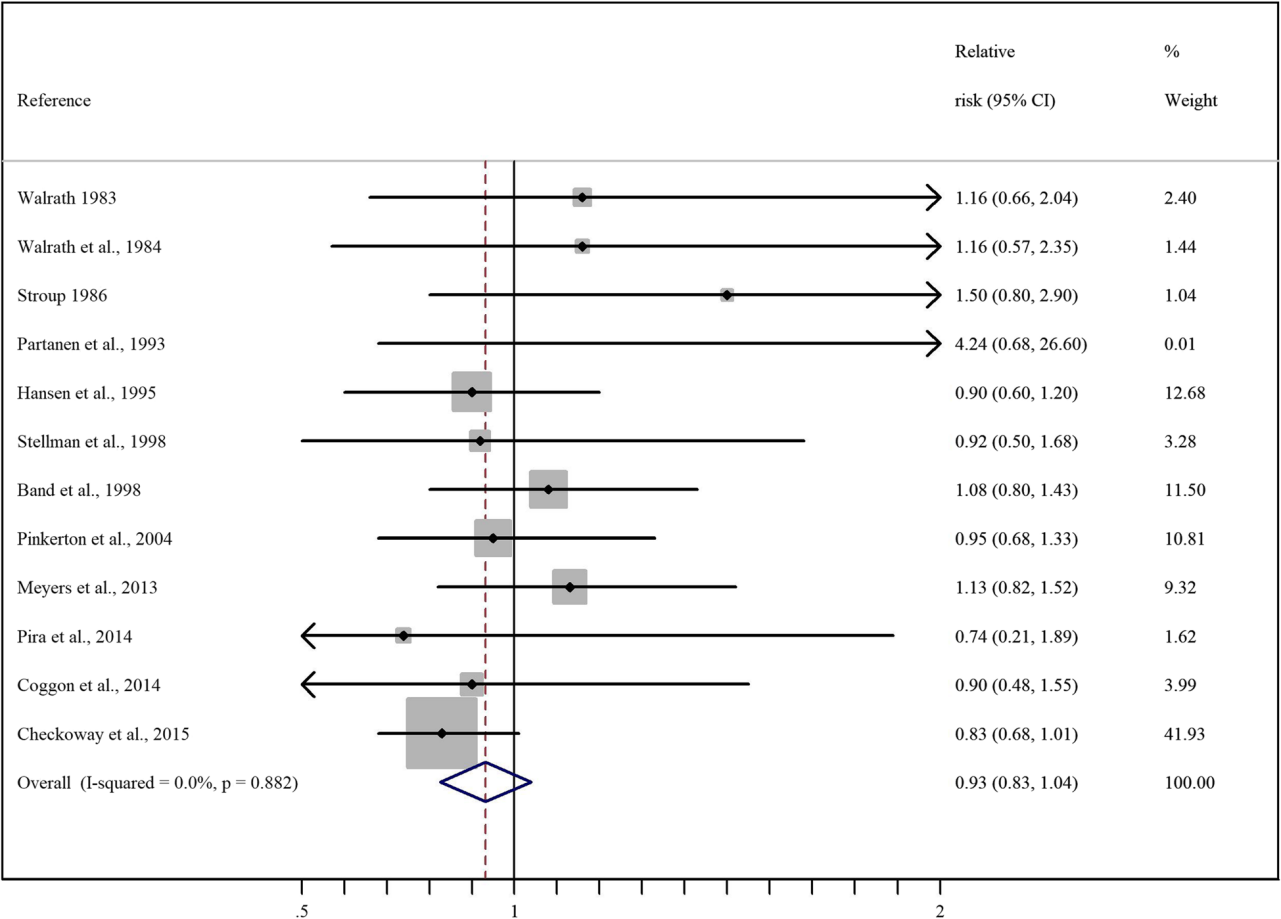
Forest plot of results of studies included in the meta-analysis. RR and 95% CIs of NHL cancer associated with Formaldehyde exposure. Horizontal lines represent 95% CIs for the study-specific RRs. The sizes of the dots for the individual studies are proportional to the study weight. The pooled RR, which was shown as a diamond, was 0.93 (0.83, 1.04). The middle of the diamond corresponds to the RR, and the width of the diamond represents the 95% CI. The arrows indicate greater or lesser confidence intervals with respect to the reported scale

The results of the individual studies are reported in Fig. 2, together with the results of the main meta-analysis, that yielded a summary RR of 0.93 (95% CI 0.83–1.04). There was no evidence of heterogeneity between studies (*p* = 0.88). The only study examining incident cases of NHL reported an SIR of 0.90 (0.60–1.20), nearly identical to the summary RR [22].

The NCI cohort [29] contributed 41.93% of the total weight of the meta-analysis, while each of the other studies contributed less than 13% of the total weight. The exclusion of the NCI cohort resulted in a meta-RR equal to 1.01 (0.95 CI 0.87–1.15). The exclusion of each of the other studies one at a time had a lesser effect, resulting in meta-RRs ranging from 0.91 to 0.94.

We conducted a further analysis of studies based on outcome definition restricted to ICD-9 codes 200,202, that resulted in a meta-RR of 0.92 (95% CI, 0.80–1.03) while the meta-RR based on the other five study with mixed definitions of outcome was 1.01 (95% CI 0.75–1.27) [ 21–24, 26, 28–29].

**Fig. 3:**
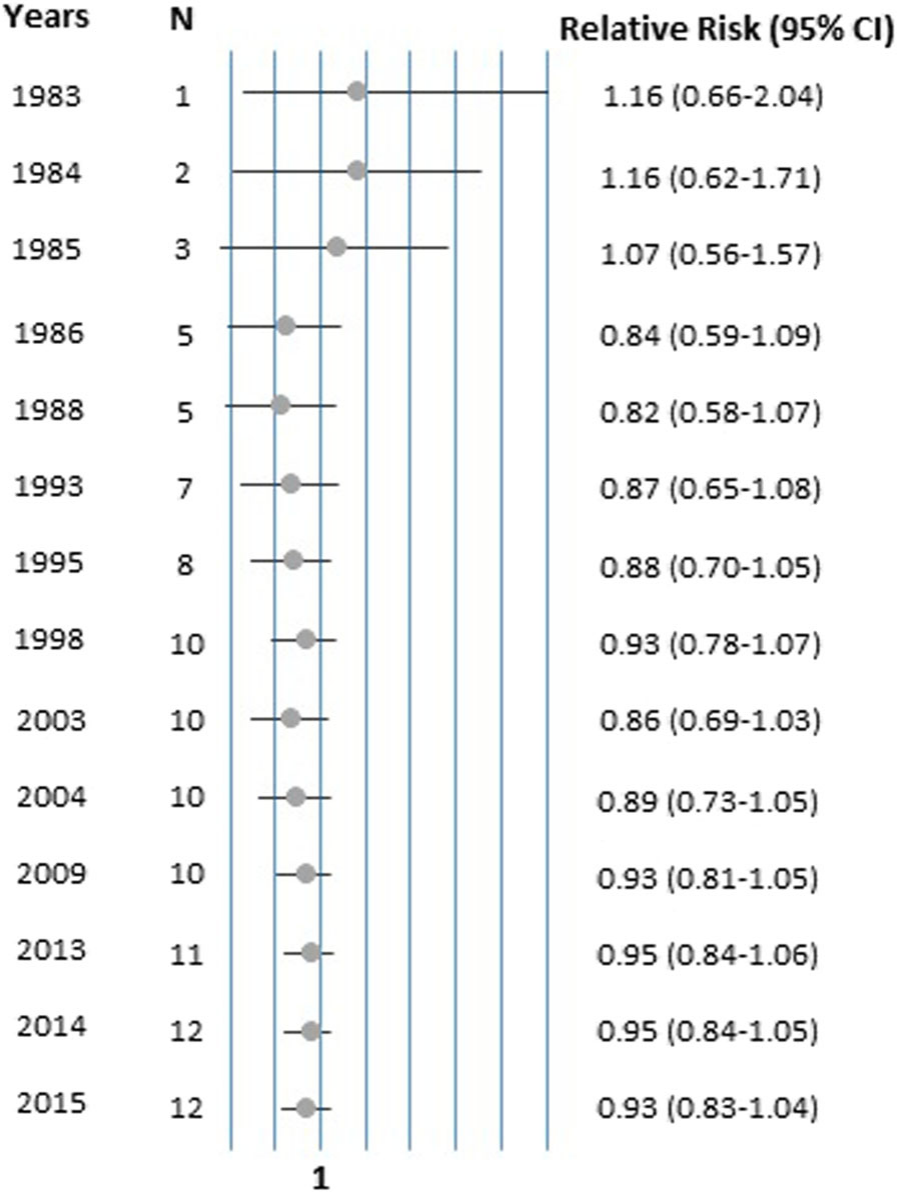
Cumulative meta-analysis according to the year of publication of the individual studies. The circles represent the RRs of the studies conducted in different years, the lines are the respective 95% CIs

The cumulative meta-analysis suggests that slightly higher RRs (all with RR ≤ 1.16 and none statistically significantly elevated) were detected based on the three earliest studies (published before 1986), while the meta-RR of studies available after 1986 ranged from 0.85 to 0.95 without a clear trend during this period (Fig. 3).

In the analysis according to level of exposure made on the basis of the data on exposure levels available from the studies included in the meta-analysis and on the type of industry, we classified as “very low” exposure the studies conducted in laminated plastic manufacturing, where formaldehyde is a by-product released from resins in the phase of machining [27], in pulp and paper mills [24] and in 265 Danish companies in which more than 1 kg of formaldehyde was used or manufactured per employee per year [22]. The meta-RR of these studies with very low exposure was 0.97 (95% CI 0.76–1.18).

Exposure was classified as “low” in studies conducted in the wood industry [21, 23] and in garment manufacturing facilities [26, 27]. The meta-RR of these studies was 1.02 (95% CI 0.80–1.24). Finally, the exposure was classified as “medium-high” in studies conducted among embalmers and anatomists [18–20], in 10 US plants producing or using formaldehyde [ 29](manufacture of formaldehyde, formaldehyde-based resins, or molding compounds, or use of formaldehyde-based resins or molding compounds, including molded plastic products, decorative laminates, photographic film, and plywood) and in six British chemical factories at a time when formaldehyde was produced or used [28]. The meta-RR of these studies was 0.87 (95% CI 0.72–1.02). There was no evidence of heterogeneity between the three groups of studies (p-heterogeneity between summary risk estimates 0.43).

**Fig. 4:**
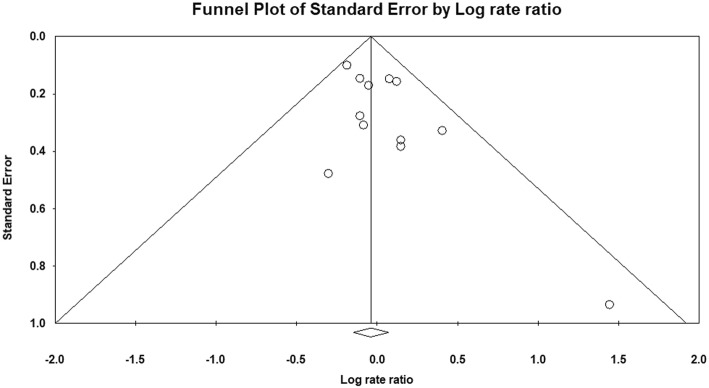
Funnel plot of studies included in the meta-analysis. Each dot represents a study; the y-axis represents study precision and the x-axis shows the study’s result

The visual assessment of the funnel plot (Fig. 4) and the result of the Egger’s test (*P* = 0.056) suggested the possibility of publication bias, with negative results of small studies apparently being missing.

Some studies carried out an assessment of the risk of NHL in relation to time-related indicators of exposure (Additional file 1: Table S2).

Results by duration of exposure were reported in a few studies. In the US garment workers’ study [26], the SMR for NHL for exposure > 10 years was 1.21 (95% CI 0.69–1.97), while in one the analyses of the NCI study [32] the RR for > 15 years of exposure was 0.98 (95% CI 0.49–1.96) and in the study of laminated plastic workers from Italy [26] the SMR for > 20 years of exposure was 0.76.

Four studies reported results by time of first formaldehyde exposure [26, 27, 35, 37]. In the study of US garment workers [26], the SMR for workers first employed before 1963 was 1.19 (95% CI 0.82–1.69), for workers first employed between 1963 and 1971 the SMR was 1.11 (95% CI 0.53–2.04) and for workers first employed after 1971 the SMR was 0.65 (95% CI 0.08–2.33) (26). In a subsequent analysis of this study [34], the SMR for other lymphatic and hematopoietic neoplasms, including NHL, was highest (SMR 1.64; 95% CI not available) among workers first exposed during 1955–1962, when potential formaldehyde exposure was highest. In the study of Italian laminated plastic workers [27] there was no trend by period of first employment. In the study of UK chemical workers [37], mortality for NHL was higher in worker employed after 1964 than in workers employed before that.

Analyses by cumulative exposure to formaldehyde were reported in several articles based on the NCI cohort study [30, 32, 33]. In particular, the RR for cumulative exposure above 5.5 ppm-years was 0.91 (95% CI 0.54–1.52), with no trend (*p* > 0.5) [33]. Analyses according to peak exposure in the same cohort also failed to detect a dose-response relationship with risk of NHL [32, 33].

## Discussion

Our meta-analysis of studies of individuals exposed to formaldehyde provided no evidence of an increased risk of NHL overall (RR 0.93; 95% CI 0.83–1.04) or by various exposure categories or characteristics. The meta-analysis comprised results from 11 independent studies with data of mortality of NHL and one that analyzed the incidence of NHL. Study populations were employed in laminated plastic manufacture, in pulp and paper mills, in the wood industry, in garment manufacturing facilities, among embalmers and anatomists and different plants producing or using formaldehyde; no differences of risk were found in different types of exposure. The limited data on time-related indicators of exposure did not find any trend indicating an increased risk of mortality for NHL with longer duration of exposure, longer ‘time since first exposure” or higher exposure level.

Because of its high water solubility and reactivity, formaldehyde is expected to exert its toxicity predominantly at the site of entry. As a result of its reactivity in target tissues, formaldehyde causes local irritation, acute and chronic toxicity and has genotoxic and cytotoxic properties [40].

Genotoxicity may play an important role in the carcinogenicity of formaldehyde in nasal tissues in humans, in addition, cellular replication in response to formaldehyde-induced cytotoxicity may promote the carcinogenic response [41]. There is controversy on the ability of formaldehyde to cause leukemia [42]. Three mechanisms were suggested by which formaldehyde could act as a leukemogenic: (i) by damaging stem cells in the bone marrow directly (ii) by damaging haematopoietic stem/progenitor cells circulating in the peripheral blood, and (iii) by damaging the primitive pluripotent stem cells present within the nasal turbinates or olfactory mucosa [13, 43–45].

NHL include a diverse group of neoplasms derived from T- and B-cells and their precursors in the lymphoid system; a genotoxic action on circulating stem cells would be consistent with the possibility that formaldehyde is a cause of lymphatic neoplasms, and one would expect that the mucosa-associated lymphatic tissue in the nasal region would be particularly at risk. There is also recognition of their inter-relatedness of these neoplasms through a common stem cell, and the malignant transformation might take place during various stages of the differentiation and maturation process of the precursor cells in bone marrow [46]. Evidence suggests that an underlying cytogenetic abnormality in an early precursor cell predisposes to subsequent mutations leading to a specific lymphohematopoietic neoplasm [47]. Assuming that the leukemogenic mechanisms mentioned above have a biologic plausibility, the possibility of a mutagenic effect of formaldehyde on circulating lymphocytes or local lymphatic tissue could not be ruled out; however, evidence on how this might occur currently is lacking.

Based on these hypothesis we decided to conduct a review of the relevant epidemiological studies investigating the relationship between occupational formaldehyde exposure and risk of NHL, but we found no evidence of an increased risk of this group of neoplasms in diverse groups of workers exposed to formaldehyde.

The main strengths of our study were the exhaustive nature of the literature search, and the focus on results with a specific definition of the phenotype that led to the exclusion of the studies that analyzed all lymphohematopoietic neoplasms (ICD 200–209), or other combinations including leukemia.

Limitations of our review reflect those of the available studies. They include the lack of quantitative assessment of exposure in most studies and the heterogeneity of the occupational exposure circumstances. We aimed at addressing the latter issue by categorizing the studies according to level of exposure, and found no difference between the groups. The cumulative meta-analysis suggests a weakly higher RR in the first three studies compared to more recent studies. Explanations for this pattern might include a higher intensity of exposure to formaldehyde (which assumes a causal relationship that is not apparent) or more likely by a greater opportunity for bias in the earliest studies. The analysis of publication bias further supports the conclusion of no association between formaldehyde exposure and risk of NHL.

Additional evidence is provided by a large, case-control study that was conducted in Montreal, Canada, during 1979–1985 [48]. This study included 3723 male cases of multiple types of cancer, including 215 cases of NHL, who were compared to 2357 cases of other cancers, and to 533 population controls. Based on detailed occupational interviews, a team of experts assessed exposure to 294 agents, including formaldehyde, by examining each work history and rating each job on a 3-point scale with regard to their confidence that exposure had actually occurred, the frequency of exposure, and the relative concentration level. Exposure was defined as ‘substantial’ when it lasted for more than 5 years at medium or higher level of frequency and concentration, and with probable or definite confidence by the experts. The prevalence of ever-exposure to formaldehyde was 15%; main occupations that included exposed subjects were carpenters and textile workers. The results were adjusted for age, ethnicity, socioeconomic status, and self/proxy status of the respondent. The OR of NHL for ever-exposure to formaldehyde was 0.8 (95% CI, derived from data reported in the publication, 0.6–1.2; 28 exposed cases); that for ‘substantial’ exposure was 1.2 (95% CI, derived from data reported in the publication, 0.5–2.7; 6 cases). These results were not included in the meta-analysis because the study did not meet the inclusion criteria; however, they are consistent with those of the meta-analysis in showing that formaldehyde exposure is not associated with increased risk of NHL.

## Conclusion

In conclusion, we found no indication of any association between various indicators of occupational exposure to formaldehyde and risk of NHL.

## Supplementary information

**Additional file 1: Table S1.** PRISMA checklist. **Table S2.** Assessment of the risk of NHL in relation to time-related indicators of exposure. **Figure S1.** Results of meta-analysis by level of exposure.

## Data Availability

The study was based entirely on previously published data. Supplementary information is available at the BMC cancer’s website.

## Abbreviations

BMI: Body mass index
CI: Confidence interval
IARC: International Agency for Research on Cancer
NHL: Non-Hodgkin lymphoma
OR: Odds ratio
PMR: Proportionate mortality ratio
RR: Relative risk
SIR: Standardized incidence ratios
SMR: Standardized mortality ratios
