# Supplementary material for: Occupational exposure to formaldehyde and risk of non hodgkin lymphoma: a meta-analysis
Source: BMC Cancer. 2019 Dec 23;19:1245. doi: 10.1186/s12885-019-6445-z (PMC6929467; doi:10.1186/s12885-019-6445-z)
Supplement: Supplementary file 1 — Additional file 1: Table S1. PRISMA checklist. Table S2. Assessment of the risk of NHL in relation to time-related indicators of exposure. Figure S1. Results of meta-analysis by level of exposure. [file 12885_2019_6445_MOESM1_ESM.docx]

**Occupational Exposure to Formaldehyde and Risk of Non Hodgkin Lymphoma: A Meta-analysis**

Table S1. PRISMA checklist.

| Section/topic | # | Checklist item | Reported on page # |
| --- | --- | --- | --- |
| TITLE | | |  |
| Title | 1 | Occupational Exposure to Formaldehyde and Risk of Non Hodgkin Lymphoma: A Meta-analysis | 1 |
| ABSTRACT | | |  |
| Structured summary | 2 | Provide a structured summary including, as applicable: background; objectives; data sources; study eligibility criteria, participants, and interventions; study appraisal and synthesis methods; results; limitations; conclusions and implications of key findings; systematic review registration number. | 2 |
| INTRODUCTION | | |  |
| Rationale | 3 | Describe the rationale for the review in the context of what is already known. | 3 |
| Objectives | 4 | Provide an explicit statement of questions being addressed with reference to participants, interventions, comparisons, outcomes, and study design (PICOS). | 4 |
| METHODS | | |  |
| Protocol and registration | 5 | Indicate if a review protocol exists, if and where it can be accessed (e.g., Web address), and, if available, provide registration information including registration number. |  |
| Eligibility criteria | 6 | Specify study characteristics (e.g., PICOS, length of follow-up) and report characteristics (e.g., years considered, language, publication status) used as criteria for eligibility, giving rationale. | 5 |
| Information sources | 7 | Describe all information sources (e.g., databases with dates of coverage, contact with study authors to identify additional studies) in the search and date last searched. | 5 |
| Search | 8 | Present full electronic search strategy for at least one database, including any limits used, such that it could be repeated. | 5 |
| Study selection | 9 | State the process for selecting studies (i.e., screening, eligibility, included in systematic review, and, if applicable, included in the meta-analysis). | 6 |
| Data collection process | 10 | Describe method of data extraction from reports (e.g., piloted forms, independently, in duplicate) and any processes for obtaining and confirming data from investigators. | 7 |
| Data items | 11 | List and define all variables for which data were sought (e.g., PICOS, funding sources) and any assumptions and simplifications made. | 7 |
| Risk of bias in individual studies | 12 | Describe methods used for assessing risk of bias of individual studies (including specification of whether this was done at the study or outcome level), and how this information is to be used in any data synthesis. | 7 |
| Summary measures | 13 | State the principal summary measures (e.g., risk ratio, difference in means). | 8 |
| Synthesis of results | 14 | Describe the methods of handling data and combining results of studies, if done, including measures of consistency (e.g., I2) for each meta-analysis. | 9 |

Table S2. Assessment of the risk of NHL in relation to time-related indicators of exposure

| **Co** | **Studies** | **Outcome** | **Variables** | **Groups** | **Risk indicator** | **Risk estimates** | |
| --- | --- | --- | --- | --- | --- | --- | --- |
|  | Walrath,1983 | ICD 200-209 | Latency period | group with<35 year of work | 121 | PMR |  |
|  |  |  |  | group with >=35 year | 121 | PMR |  |
|  |  |  | Age at the first license | <30 years | 138 | PMR |  |
|  |  |  |  | >30 years | 93 | PMR |  |
|  | Band, 1997 | ICD 200-202 | Work duration | <15 years and less than 15 years since first employed | 1.58 (0.27-4.96) | SMR (95% CI) |  |
|  |  |  |  | <15 years and more than 15 years since first employed | 1.90 (0.33-5.98) | SMR (95% CI) |  |
|  |  |  |  | > 15 years and more than 15 years since first employed | 1.70 (0.89-2.96) | SMR (95% CI) |  |
|  | Meyers, 2013 | NHL | Year of first exposure | Prior to 1963 | 1.19 (0.82-1.69) | SMR (95% CI) |  |
|  |  |  |  | 1963 - 1970 | 1.11 (0.53-2.04) | SMR (95% CI) |  |
|  |  |  |  | 1971 or later | 0.65 (0.08-2.33) | SMR (95% CI) |  |
|  |  |  | Time since first exposure | <10 years | 0.52 (0.01-2.91) | SMR (95% CI) |  |
|  |  |  |  | 10-19 years | 0.70 (0.14-2.05) | SMR (95% CI) |  |
|  |  |  |  | >20 years | 1.22 (0.87-1.67) | SMR (95% CI) |  |
|  |  |  | Duration of Formaldehyde Exposure | <3 years | 1.16 (0.68-1.86) | SMR (95% CI) |  |
|  |  |  |  | 3-9 years | 0.99 (0.49-1.77) | SMR (95% CI) |  |
|  |  |  |  | 10 years | 1.21 (0.69-1.97) | SMR (95% CI) |  |
|  | Pira, 2014 | Lymphoma | Duration of exposure | <10 | 0.42 | SMR |  |
|  |  |  |  | 10-19 | 1.29 | SMR |  |
|  |  |  |  | >20 | 0.76 | SMR |  |
|  |  |  | Age at first exposure | <25 | 0.88 | SMR |  |
|  |  |  |  | 25-34 | 0 | SMR |  |
|  |  |  |  | >35 | 1.19 | SMR |  |
|  |  |  | Time since first exposure | <20 | 0 | SMR |  |
|  |  |  |  | 20-29 | 0.77 | SMR |  |
|  |  |  |  | >30 | 1.3 | SMR |  |
|  |  |  | Time since last exposure | During exposure | 0 | SMR |  |
|  |  |  |  | 1– <10 | 0 | SMR |  |
|  |  |  |  | >10 | 1.47 | SMR |  |
|  |  |  | Period at first exposure | Before 1970 | 1.29 | SMR |  |
|  |  |  |  | 1970–1979 | 0 | SMR |  |
|  |  |  |  | 1980 or after | 0 | SMR |  |
| UK | Coggon,2014 | NHL | Exposure to Formaldehyde | high (> 2.0 ppm) | 0.90 (0.48, 1.55) | SMR (95% CI) |  |
|  |  |  |  | all subjects | 1.06 (0.79, 1.38) | SMR (95% CI) |  |
|  |  |  |  | background (<0.1ppm) | 0.31 (0.06, 0.91) | SMR (95% CI) |  |
|  |  |  |  | low/moderate (0.1-2.0 ppm) | 1.47 (0.82, 2.43) | SMR (95% CI) |  |
| NCI | Checkoway, 2015 | NHL | Cumulative Exposure to Formaldehyde | 0–<0.5 ppm/years | 1.0 (referent) | HR† (95% CI) |  |
|  |  |  |  | 0.5–<2.5 | 0.96 (0.63–1.46) | HR† (95% CI) |  |
|  |  |  |  | ≥2.5 | 0.77 (0.51–1.16) | HR† (95% CI) | p trend 0.22 |
| NCI | Hauptmann, 2003 | NHL | Peak exposure to formaldehyde | 0 ppm | 1.12 (0.38 to 3.31) | RR (95% CI) |  |
|  |  |  |  | 0.1–1.9 ppm | 1.0 referent | RR (95% CI) |  |
|  |  |  |  | 2.0–3.9 ppm | 1.39 (0.67 to 2.91) | RR (95% CI) |  |
|  |  |  |  | >4.0 ppm | 1.23 (0.59 to 2.55) | RR (95% CI) | p trend 0.604 |
|  |  |  | Average intensity of exposure | 0 ppm | 1.02 (0.36 to 2.86) | RR (95% CI) |  |
|  |  |  |  | 0.1–0.4 ppm | 1.00 (Referent) | RR (95% CI) |  |
|  |  |  |  | 0.5–0.9 ppm | 1.33 (0.65 to 2.71) | RR (95% CI) |  |
|  |  |  |  | > 1.0 ppm | 0.98 (0.43 to 2.20) | RR (95% CI) | p trend 0.69 |
|  |  |  | Cumulative exposure to formaldehyde | 0 ppm/years | 0.82 (0.29 to 2.34) | RR (95% CI) |  |
|  |  |  |  | 0.1–1.4 | 1.00 (Referent) | RR (95% CI) |  |
|  |  |  |  | 1.5–5.4 | 0.53 (0.22 to 1.31) | RR (95% CI) |  |
|  |  |  |  | >5.5 | 0.92 (0.45 to 1.88) | RR (95% CI) | p trend 0.97 |
|  |  |  | Duration of exposure to formaldehyde | 0 years | 0.86 (0.29 to 2.49) | RR (95% CI) |  |
|  |  |  |  | 0.1–4.9 | 1.00 (Referent) | RR (95% CI) |  |
|  |  |  |  | 5.0–14.9 | 0.56 (0.21 to 1.49) | RR (95% CI) |  |
|  |  |  |  | > 15 years | 0.98 (0.49 to 1.96) | RR (95% CI) | p trend 0.61 |
| NCI | Beane Freeman, 2009 | NHL | Peak exposure to formaldehyde | 0 ppm | 1.06 (0.53 to 2.14) | RR (95% CI) |  |
|  |  |  |  | 0.1–1.9 ppm | 1.00 (Referent) | RR (95% CI) |  |
|  |  |  |  | 2.0–3.9 ppm | 1.08 (0.65 to 1.78) | RR (95% CI) |  |
|  |  |  |  | >4.0 ppm | 0.91 (0.55 to 1.49) | RR (95% CI) | p trend >0.5 |
|  |  |  | Average intensity of exposure | 0 ppm | 1.08 (0.55 to 2.12) | RR (95% CI) |  |
|  |  |  |  | 0.1–0.4 ppm | 1.00 (Referent) | RR (95% CI) |  |
|  |  |  |  | 0.5–0.9 ppm | 1.20 (0.73 to 1.96) | RR (95% CI) |  |
|  |  |  |  | > 1.0 ppm | 0.71 (0.39 to 1.32) | RR (95% CI) | p trend >0.5 |
|  |  |  | Cumulative exposure | 0 ppm/years | 0.94 (0.46 to 1.86) | RR (95% CI) |  |
|  |  |  |  | 0.1–1.4 | 1.00 (Referent) | RR (95% CI) |  |
|  |  |  |  | 1.5–5.4 | 0.58 (0.31 to 1.06) | RR (95% CI) |  |
|  |  |  |  | >5.5 | 0.91 (0.54 to 1.52) | RR (95% CI) | p trend >0.5 |
| NCI | Blair, 1986 | Lymphoma and reticulosarcoma (200) | Cumulative exposure to formaldehyde | 0 ppm/years | 203 (42-592) | SMR (95% CI) |  |
|  |  |  |  | <0.5 | 34 (4-121) | SMR (95% CI) |  |
|  |  |  |  | 0.51-5.5 | 63 (17-160) | SMR (95% CI) |  |
|  |  |  |  | >5.5 | 56 (7-202) | SMR (95% CI) |  |
|  |  | Other lymphatic tissue (202, 203, 208) | Cumulative exposure to formaldehyde | 0 ppm/years | 135 (16-487) | SMR (95% CI) |  |
|  |  |  |  | <0.5 | 148 (64-291) | SMR (95% CI) |  |
|  |  |  |  | 0.51-5.5 | 95 (35-207) | SMR (95% CI) |  |
|  |  |  |  | >5.5 | 100 (27-255) | SMR (95% CI) |  |
| UK | Gardner, 1993 | NHL | Year of First Exposure | before 1965 | 93 (48-163) | SMR (95% CI) |  |
|  |  |  |  | after 1964 | 188 (76-388) | SMR (95% CI) |  |
| NIOSH | Stayner, 1985 | Other lymphatic and hematopoietic | Duration of Exposure and Latency | Latency < 10 years and duration <10 years | 0 | PMR |  |
|  |  |  |  | Latency > 10 years and duration <10 years | 0 | PMR |  |
|  |  |  |  | Latency > 10 years and duration >10 years | 761 | PMR |  |
| NIOSH | Stayner, 1988 | Other lymphatic and hematopoietic | Year of First Exposure | 1955-1962 | 164 | SMR |  |
|  |  |  |  | 1963-1970 | 0 | SMR |  |
|  |  |  |  | 1971-1 978 | 1 | SMR |  |


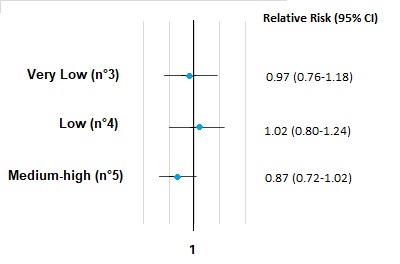


Figure S1. Results of meta-analysis by level of exposure
